# Anti-Cancer Activity of Derivatives of 1,3,4-Oxadiazole

**DOI:** 10.3390/molecules23123361

**Published:** 2018-12-18

**Authors:** Teresa Glomb, Karolina Szymankiewicz, Piotr Świątek

**Affiliations:** Department of Chemistry of Drugs, Faculty of Pharmacy, Wroclaw Medical University, Borowska 211, 50-556 Wroclaw, Poland; teresa.glomb@umed.wroc.pl (T.G.); karolina_2805@o2.pl (K.S.)

**Keywords:** 1,3,4-oxadiazole, anti-proliferative activity, anti-cancer drugs

## Abstract

Compounds containing 1,3,4-oxadiazole ring in their structure are characterised by multidirectional biological activity. Their anti-proliferative effects associated with various mechanisms, such as inhibition of growth factors, enzymes, kinases and others, deserve attention. The activity of these compounds was tested on cell lines of various cancers. In most publications, the most active derivatives of 1,3,4-oxadiazole exceeded the effect of reference drugs, so they may become the main new anti-cancer drugs in the future.

## 1. Introduction

Around the world research is underway to find new anti-cancer drugs. The constant increase in the incidence [1], the numerous side effects of the drugs currently in use [2], as well as the developing resistance of tumours to drugs [3] force the continuous search for new molecules with a safer effect profile. New synthetic anti-cancer compounds are most often heterocyclic derivatives, whereby structures containing a 1,3,4-oxadiazole ring constitute a group of compounds with exceptionally high cytostatic potential.

**Figure 1:**
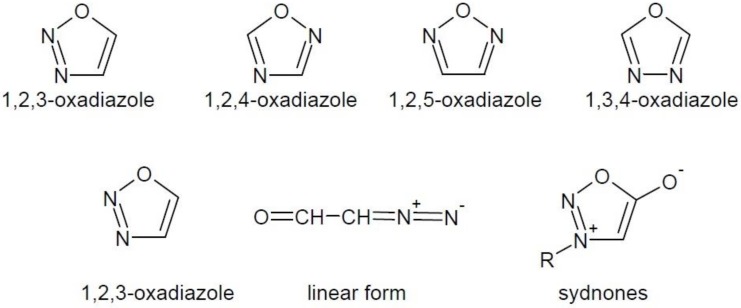
Isomeric forms of oxadiazole and modifications of unstable ring of 1,2,3-oxadiazole.

Oxadiazoles are five-membered heterocyclic compounds containing two nitrogen atoms and one oxygen atom in their structure. They occur in several isomeric forms (Figure 1).

**Figure 2:**
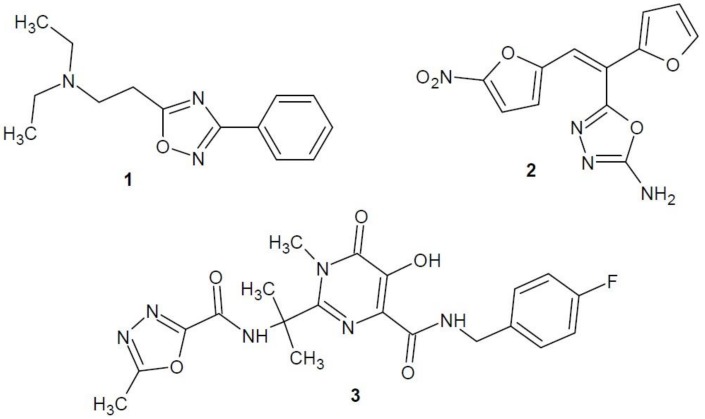
Drugs with oxadiazole core.

The 1,2,3-oxadiazole ring is unstable and is tautomerised to diazo-ketone linear form. It does not occur in the free form, but in rare mesoionic forms, called sydnones [4] (Figure 1). The other oxadiazole isomers are well known and occur in the structure of many drugs, e.g., antitussive oxolamine (**1**), antimicrobial furamizole (**2**), antiviral raltegravir (**3**) and others (Figure 2).

Particularly noteworthy are the derivatives of 1,3,4-oxadiazole. The presence of the 1,3,4-oxadiazole ring affects the physicochemical and pharmacokinetic properties of the compounds in which it is present. Compared to other isomeric oxadiazoles, 1,3,4-derivatives show better metabolic stability, water solubility and lower lipophilicity. The 1,3,4-oxadiazole ring also acts as a bioisosteres for carbonyl containing compounds such as esters, amides and carbamates. Oxadiazole ring is used as a substantial part of the pharmacophore, which have the ability to engage with ligand. In some cases, it acts like a flat aromatic linker to provide the appropriate orientation of the molecule [5]. 

There are numerous literature reports confirming the multidirectional effect of compounds containing the 1,3,4-oxadiazole ring in its structure. Derivatives of this type have antibacterial [6], antimalarial [7], anti-inflammatory [8], antidepressive [9], anticancer [10], analgesic [11] and antiviral effect [12,13]. In view of the constantly increasing incidence of various types of cancer, research on the anti-cancer properties of 1,3,4-oxadiazole derivatives seems to be of particular interest. The oxadiazole derivatives discussed in this publication may act cytostatically through various mechanisms related to the inhibition of growth factors, enzymes, kinases and others. 

## 2. Anti-Proliferative Effects of 1,3,4-Oxadiazole Derivatives

### 2.1. Epidermal Growth Factor Receptor Inhibitors

Growth factors and their transmembrane receptors play a very important role in the normal functioning of cells. These receptors have internal activity of tyrosine kinase enzyme, thus catalysing phosphorylation of proteins associated with signalling intracellular processes, e.g., proliferation, differentiation, and cell apoptosis. One of these receptors is EGFR—epidermal growth factor receptor also known as HER1 (erbB1) and HER2 receptor (erbB2). Their improper activation or overexpression leads to uncontrolled cell growth and thus to the development of cancer. They also play a role in metastasis and angiogenesis of neoplasms, and their inhibition leads to tumour regression. For this reason, these receptors are often used in targeted cancer therapy [14,15,16].

**Figure 3:**
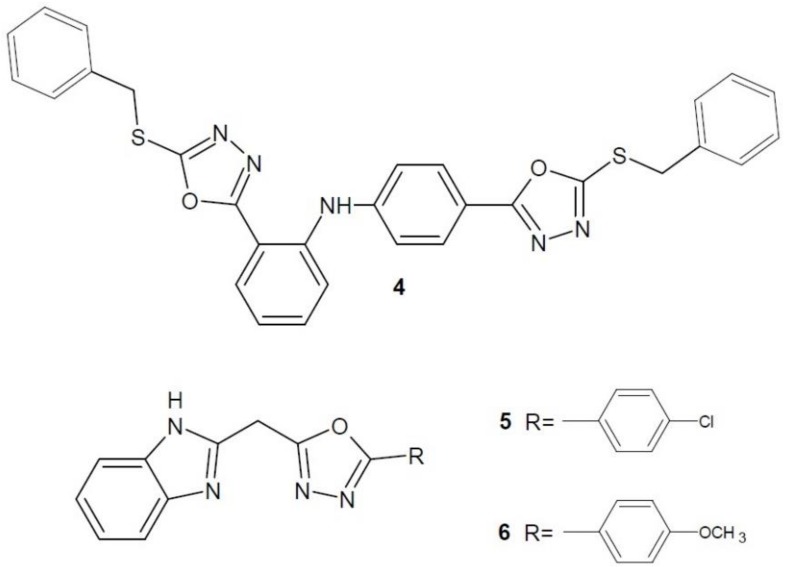
1,3,4-Oxadiazole derivatives with activity of epidermal growth factor receptor inhibitors.

Researchers under the direction of Abou-Seri (2010) received a number of bis-5-mercapto-1,3,4-oxadiazole derivatives. The best anti-proliferative properties against MCF-7 breast cancer cell line were demonstrated by the most lipophilic, dibenzyl derivative **4** (Figure 3). Additional studies of compound **4** for EGFR tyrosine kinase showed significant activity compared to the reference lapatinib [17].

Akhtar et al. (2017) developed a series of new benzimidazole derivatives of 1,3,4-oxadiazole and tested their cytotoxicity to five cancer cell lines – breast cancer (MCF-7, MDA-MB231), skin cancer (HaCaT), liver cancer (HepG2) and lung cancer (A549). Compounds **5** and **6** (Figure 3) had a stronger cytotoxic effect on breast cancer cells (MCF-7) than the reference compound, i.e., 5-fluorouracil. The obtained compounds were also tested for binding to EGFR and HER2 receptors. It was confirmed that their binding is analogous to the anti-cancer drug inhibiting tyrosine kinase—erlotinib [18].

### 2.2. Vascular Endothelial Growth Factor Receptor Inhibitors

Angiogenesis, i.e., the formation of new blood vessels, is a physiological process without which the functioning of tissues would be impossible. On the other hand, pathological angiogenesis is the cause of the spread of diseases, e.g., cancer. Vascular endothelial growth factor (VEGF) is the cytokine responsible for this process. It occurs in several isoforms and has three types of transmembrane receptors with tyrosine kinase activity on endothelial cells: VEGFR-1, VEGFR-2 and VEGFR-3. The VEGFR-2 receptor is located on the endothelial cells of blood vessels and is therefore involved in angiogenesis through participation in proliferation, migration and differentiation of these cells [19,20]. Neovascularisation is a key process during carcinogenesis and cancer metastases. Therefore, interrupting the signalling pathway to the VEGFR-2 receptor is an attractive target for cancer therapy. 

**Figure 4:**
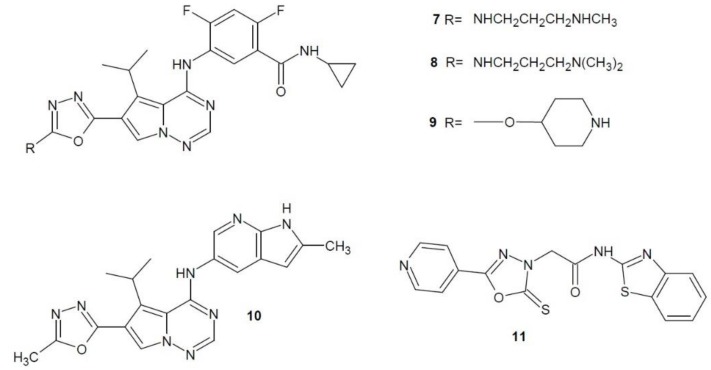
1,3,4-Oxadiazole derivatives with activity of vascular endothelial growth factor receptor inhibitors.

In 2008, Cai et al. examined the inhibitory activity of pyrrolotriazine derivatives of 1,3,4-oxadiazole against the VEGFR-2 receptor, and the most active derivatives were subjected to human umbilical vein endothelial cells (HUVEC) proliferation assay. The highest inhibitory potential was shown by compounds **7**, **8**, **9** (Figure 4). Compound **9** was also tested in vivo on human lung cancer cells (L2987) transplanted to mice, which confirmed its anticancer activity [21].

Ruel et al. (2008) tested the inhibitory effects of other pyrrolotriazine derivatives of 1,3,4-oxadiazole on the VEGFR-2 receptor. The most active derivative was compound **10** (BMS-645737) (Figure 4) which was also pre-clinically tested in vivo on various types of cancers in a heterogeneous model. It showed the highest anti-cancer activity for human lung cancer cells (L2987) [22].

Bhanushali et al. (2017) developed a series of 5-pyridin-4-yl-2-thioxo-1,3,4-oxadiazol-3-yl derivatives and tested them as potential VEGFR-2 inhibitors. The most active compound **11** (Figure 4) showed a significant inhibition of angiogenesis in the CAM sample (Chick Chorioallontoic Membrane) compared to the reference sorafenib. In addition, the conducted in vivo tests have also shown its significant activity. Furthermore, it was examined that it had a high inhibitory potential against VEGFR-2 tyrosine kinase [23].

### 2.3. Endothelin Receptor Antagonists

Endothelin 1 (ET-1) is a vasoactive peptide that binds to two types of antagonistic receptors. Activation of the ET_A_ receptor by endothelin-1 results in the proliferation of cells and increases their survival rate, whereas activation of the ET_B_ receptor results in the apoptosis of cells and decreases the amount of ET-1. ET_A_ receptor overexpression occurs in many types of cancer. By inhibiting the activation of the ET_A_ receptor, while maintaining the activity of the ET_B_ receptor, an anti-cancer effect [24] is achieved. 

**Figure 5:**
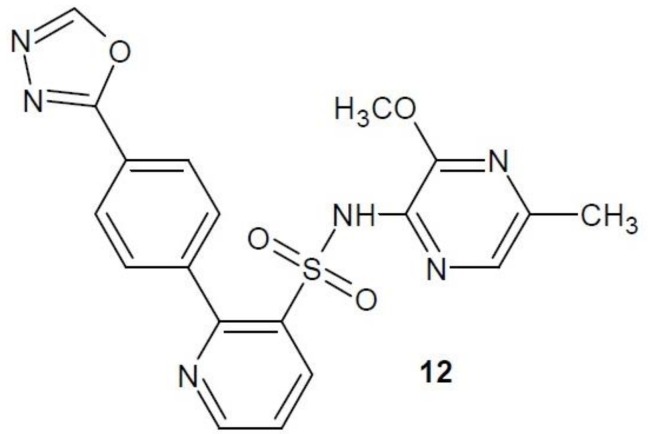
Zibotentan with activity of endothelin receptor antagonist.

Zibotentan (**12**) (Figure 5) is a selective ET_A_ receptor inhibitor tested in vitro for efficacy in colorectal cancer. It showed high anti-cancer activity [25]. However, the third stage of clinical trials of zibotentan in the treatment of castration-resistant prostate cancer (CRPC) was unsuccessful. Further tests of its effectiveness in other types of cancers are planned [26,27].

### 2.4. Focal-Adhesion Kinase Inhibitors

Focal-adhesion kinase (FAK) is a cytoplasmic tyrosine kinase and its main function is to transmit a signal from integrin receptors or growth factor receptors to the intracellular protein cascade. FAK protein takes part in cell cycle regulation, adhesion, migration and cell apoptosis. Enhanced FAK signalling may cause uncontrolled proliferation or migration of cells, which has been observed in the process of cancer development and progression [28]. This explains the use of focal-adhesion kinase inhibitors as anti-cancer drugs. 

**Figure 6:**
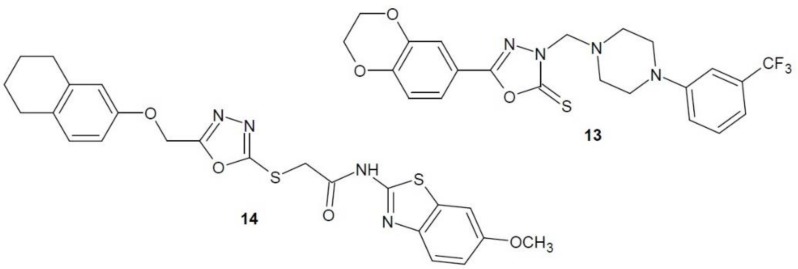
1,3,4-Oxadiazole derivatives with activity of focal-adhesion kinase inhibitors.

In 2017, Sun et al. published the results of research on 1,3,4-oxadiazole derivatives as FAK inhibitors. Several phenylpiperazine derivatives of 1,3,4-oxadiazole were studied, the most effective of which seems to be 3-trifluoromethyl-piperazine **13** (Figure 6). Four cancer cell lines were used in cytotoxicity testing: liver cancer (HepG2), cervical cancer (HeLa), colorectal cancer (SW1116) and stomach cancer (BGC823). Compound **13** was the most effective in inhibiting the growth of liver cancer cells (HepG2) compared to the reference compound 5-fluorouracil. The influence of compound **13** on tyrosine kinase activity was also studied. Among all the derivatives obtained, it showed the highest FAK inhibitory activity [29].

In 2018, another team of scientists published the results of research on thiazole and benzothiazole derivatives of 1,3,4-oxadiazole as FAK inhibitors. A number of compounds were tested on three cell lines: human lung cancer (A449), rat glioma (C6) and mouse embryonic fibroblasts (NIH/3T3). The most active derivative **14** (Figure 6) showed a stronger anti-proliferative effect than the cisplatin reference drug against all cell lines. In addition, the compound was characterised by a strong FAK inhibitory activity [30].

### 2.5. Histone Deacetylase Inhibitors

Gene expression is regulated by posttranslational modification of histone proteins, e.g., acetylation, methylation, phosphorylation. Acetylation and deacetylation are regulated by two groups of opposing enzymes: histone acetyltransferases (HAT) and histone deacetylases (HDAC), leading to gene transcription or silencing, respectively. The HDAC family contains 18 enzymes involved in the deacetylation and regulation of gene expression. Scientists observed that HDAC overexpression is associated with carcinogenesis and tumour progression, therefore inhibition of the enzyme is one of the mechanisms of anti-cancer drugs. HDAC inhibitors cause cancer cell death in many ways, including apoptosis, autophagy, inhibition of DNA repair and control of angiogenesis [31,32,33].

**Figure 7:**
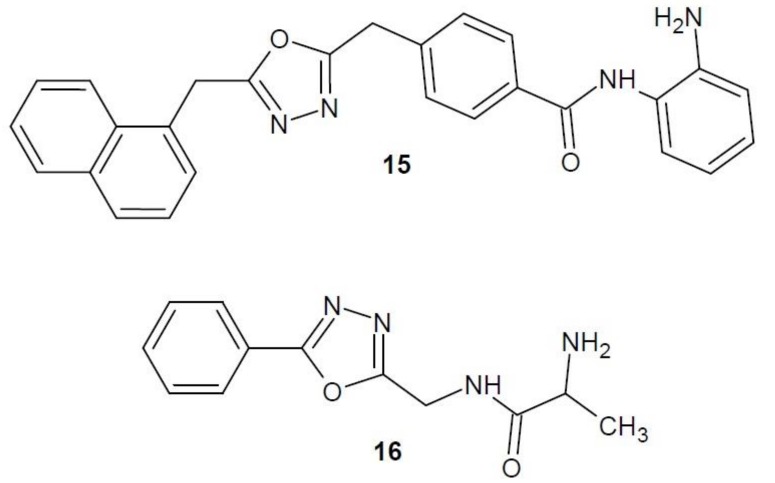
1,3,4-Oxadiazole derivatives with activity of histone deacetylase inhibitors.

In 2014, a publication by Valente et al. on new derivatives of 1,3,4-oxadiazole as inhibitors of histone deacetylase was published. Of all the compounds obtained, the most extensive effect was shown by compound **15** (Figure 7). Its anti-proliferative activity against many cell lines was studied, and the most sensitive to its effects were colon adenocarcinoma cell lines (SW620) and all of the studied acute myeloid leukaemia cell lines (U937, HL60, HEL, KG1 and MOLM13). Studies also showed that compound **15** strongly inhibited HDAC histone deacetylases as compared to the reference compound—vorinostat [31].

An Indian research team (2016–2017) combined a 1,3,4-oxadiazole molecule with amino acids, alanine or glycine, and studied the effect of the resulting hybrids on the activity of HDAC8, one of the enzymes of the histone deacetylase family. The most active structure turned out to be the derivative **16** (Figure 7). The effectiveness of compound **16** in inhibiting cancer cell proliferation and its mechanism of action have been studied in more detail in vitro. The results confirmed that compound **16** has an inhibitory effect on breast cancer cell growth (MCF-7 and MDA-MB-231), as well as high selectivity against HDAC8. The researchers have also developed a molecular mechanism of action of derivative **16** in breast cancer cells. This molecule induces the internal cell apoptosis through a series of consecutive processes. Selective inhibition of HDAC8 enzyme leads to the activation of p53 protein and change of Bax/Bcl2 protein ratio (decreased expression of Bcl-2 antiapoptotic protein, without influence on the expression of proapoptotic Bax protein). Subsequently, the potential of mitochondrial membrane decreases, cytochrome c is released, thus activating caspase 3 and 9 and leading to the cutting of the PARP enzyme and, eventually, to apoptosis [34,35].

### 2.6. Methionine Aminopeptidase Inhibitors

Methionine aminopeptidase (MetAP) is a protein that plays an important role in the regulation of posttranslational processes and protein synthesis. It is an enzyme, metalloprotease, that is responsible for the removal of methionine (amino acid from the N-end of new proteins), which is necessary for their further modification. There are two forms of the enzyme: MetAP1 and MetAP2. Higher concentration of MetAP2 in cancer cells compared to normal cells suggests that this enzyme plays an important role in cell proliferation and tumour growth [36]. Therefore, research on MetAP inhibitor compounds as potential cancer drugs is constantly being developed.

**Figure 8:**
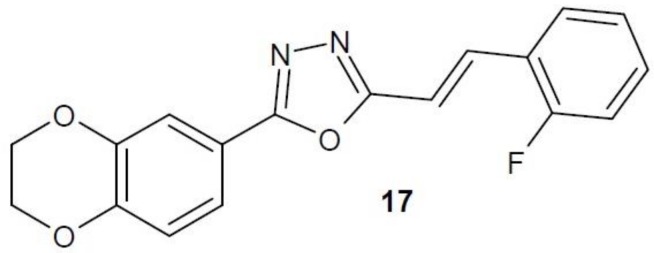
1,3,4-Oxadiazole derivative with activity of methionine aminopeptidase inhibitor.

A team of Chinese scientists designed and synthesised a series of 1,3,4-oxadiazole derivatives with 1,4-benzodioxane substituent. In vitro studies on anti-cancer activity showed that compound **17** (Figure 8) strongly inhibited MetAP2 activity and its anti-proliferative effect was confirmed by studies on human umbilical vein endothelial cells (HUVEC) [37].

### 2.7. NF-κB Inhibitors

NF-κB (nuclear factor κB) is one of the most popular inflammatory mediators in many diseases, including cancer. It is a nuclear transcription factor, activated by inflammatory processes, DNA damage, stress and other factors. NF-κB regulates the expression of cytokines and adhesion factors responsible for intercellular interactions. Signalling pathways of NF-κB are canonical and noncanonical. Many types of cancer cause excessive activation of the NF-κB factor, thus leading to the expression of genes responsible for the proliferation and protecting the cell from factors causing its death by apoptosis [38,39,40]. For this reason, NF-κB pathway inhibitors are promising in the treatment of cancer. Moreover, inhibiting NF-κB activation can result in the reversal of chemoresistance. Therefore NF-κB inhibitors have potential as adjuvant therapy [41,42].

**Figure 9:**
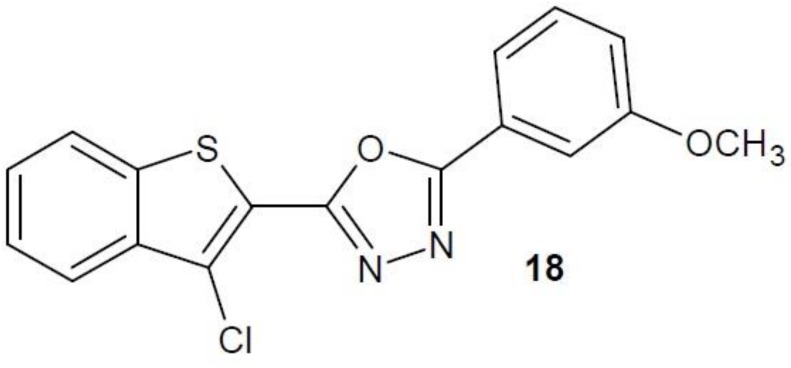
1,3,4-Oxadiazole derivative with activity of nuclear factor κB inhibitor.

In March 2018, Mohan’s work on new 1,3,4-oxadiazole derivatives and their anti-cancer activity based on the level of NF-κB activity in hepatocellular carcinoma cells (HCC) was published. Among the obtained compounds, the highest potential was shown by derivative **18** (Figure 9). The activity of the compound **18** was determined by the flow cytometry method and by studying protein phosphorylation level of the NF-κB signalling pathway in HCC cells. Studies showed that the derivative obtained has a dose- and time-dependent anti-proliferative effect and, in addition, causes cell apoptosis, most likely through the activation of caspase-3 [43].

### 2.8. Poly(ADP-ribose) Polymerase Inhibitors

PARP-1 (poly(ADP-ribose) polymerase) is the best known enzyme of the PARP family. It plays an important role in many biological processes such as gene transcription, DNA repair and cell death regulation. An increase in the concentration of this enzyme is observed in various types of cancer. Its overexpression influences the process of angiogenesis and progression of tumours, as well as increases tissue inflammation. It is a subject of preclinical and clinical studies as an attractive target for new anti-cancer agents. PARP-1 inhibitors such as olaparib, veliparib and rucaparib have been approved for the treatment of ovarian, breast, prostate and pancreatic tumours [44].

**Figure 10:**
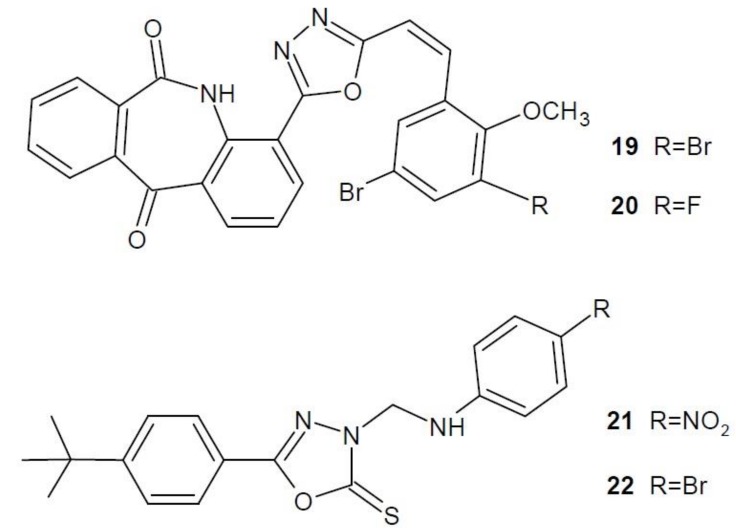
1,3,4-Oxadiazole derivatives with activity of poly(ADP-ribose) polymerase inhibitors.

In 2017 Yadava et al. studied a new series of Mannich bases—2-thioxo-1,3,4-oxadiazole analogues as PARP inhibitors. In the series of derivatives obtained, compounds **19** and **20** (Figure 10) showed stronger proapoptotic activity. The study was conducted on four cancerous cell lines: cervical (HeLa), pancreatic (Panc), breast (MCF-7) and glioblastoma (U-87). Compounds **19** and **20** showed the highest activity against ovarian cancer cells (HeLa). Moreover, they were much more selective towards all cell lines than doxorubicin, used as a reference drug. The mechanism of action of compounds **19** and **20** was also examined in more detail. They influence the apoptosis and inhibition of the cell cycle by activating caspase-3, arresting the cell in the G2-M phase of the cycle, as well as through condensation of chromatin and cutting of PARP. In addition, there is an increased expression of proapoptotic factor Bax with decreased expression of antiapoptotic factor Bcl-2 [45].

Studies by He et al. from 2018 prove that 5*H*-dibenzo[b,e]azepin-6,11-dione derivatives containing the 1,3,4-oxadiazole ring have a PARP-1 inhibitory effect. The effect of these compounds on the growth of ovarian cancer cells (OVCAR-3) was studied. Compounds **21** and **22** (Figure 10) showed the strongest anti-proliferation activity, even greater than the reference compound rucaparib. In order to verify PARP-1 inhibition by compounds **21** and **22**, the activity inhibiting this enzyme was examined. The results suggest that these are strong inhibitors, with compound **22** having practically the same activity as the reference drug [46]. 

### 2.9. Telomerase Inhibitors

Telomeres are specialised structures at the ends of chromosomes consisting of repeating hundreds or even thousands of time sequences of TTAGGG. In most somatic cells, the length of the telomere is shortened during DNA replication; it is the so-called mitotic clock controlling the number of cell divisions. When telomeres reach critically short lengths, this results in genome instability and cell apoptosis [47]. Unlike normal cells, in tumour cells, the length of the telomere is stabilised and renewed by an enzyme synthesising telomeres *de novo*—telomerase. Therefore, their division is unlimited. Progression of the tumour can be interrupted by shortening the telomere, which is possible thanks to the use of telomerase inhibitors [48].

**Figure 11:**
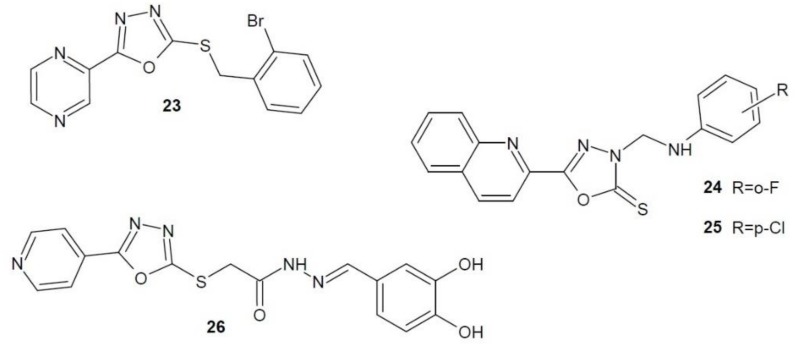
1,3,4-Oxadiazole derivatives with activity of telomerase inhibitors.

Researchers from China (2012) synthesised a number of 1,3,4-oxadiazole derivatives containing the pyrazine group. They were examined on four cancer cell lines: liver (HEPG2), colorectal (SW1116), cervical (HELA) and stomach cancer (BGC823). The most active derivative **23** (Figure 11) showed strong anti-proliferative activity against SW1116 cells, comparable to the reference 5-fluorouracil. The activity of derivative **23** as a telomerase inhibitor was also studied. It had a stronger effect than staurosporin [49].

Sun et al. (2013) obtained and studied 1,3,4-oxadiazole derivatives containing quinoline group. They were examined on three cancer cells lines: liver cancer (HepG2), stomach cancer (SGC-7901) and breast cancer (MCF-7). The most active derivatives **24** and **25** (Figure 11) showed anti-proliferative effects even 20 times higher than 5-fluorouracil. In addition, their action as a telomerase inhibitor was much stronger than the reference staurosporin [50].

Zhang et al. (2014) examined the inhibitory effect on telomerase of pyridine 1,3,4-oxadiazole analogues [51]. Among the derivatives obtained, compound **26** (Figure 11) showed the strongest anti-cancer activity against four different cancer cells lines - liver cancer (HEPG2), breast cancer (MCF7), colorectal cancer (SW1116) and stomach cancer (BGC823), even stronger than 5-fluorouracil, the reference compound in the study. The activity of derivative **26** as a telomerase inhibitor was also studied. Again, compound **26** showed high inhibitory potential with regard to this enzyme, higher than the comparative drug—staurosporine.

### 2.10. Thymidine Phosphorylase Inhibitors

Thymidine phosphorylase (TP) is an enzyme otherwise known as platelet-derived endothelial cell growth factor (PD-ECGF). The effect of this enzyme consists in the reversible conversion of thymidine to thymine. During the degradation of pyrimidine nucleoside, namely thymidine, thymine and 2-deoxy-d-ribose 1-phosphate are formed. This is followed by complete dephosphorylation and formation of 2-deoxy-d-ribose with in vitro chemotactic and in vivo angiogenic activity by stimulating VEGF (vascular endothelial growth factor) secretion. Thymidine phosphorylase occurs in healthy tissues of the body, and in higher concentrations also in cancerous tissues. Physical and chemical stress causes activation of the enzyme in the cancer tissue, which leads to an increase in the concentration of 2-deoxy-d-ribose, causing the formation of new vessels and, consequently, the progression of tumours [52]. It is therefore reasonable to use TP inhibitors as anti-cancer drugs.

**Figure 12:**
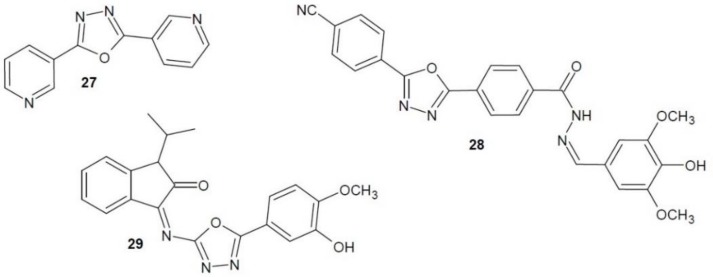
1,3,4-Oxadiazole derivatives with activity of thymidine phosphorylase inhibitors (part 1).

Khan et al. (2013) developed a series of 2,5-substituted 1.3.4-oxadiazoles and tested them for thymidine phosphorylase activity. The most active derivative was compound **27** with a 3-pyridyl substituent in positions 2 and 5 (Figure 12). Its activity as an enzyme inhibitor was comparable to that of the reference 7-deazaxanthin [53].

Ullah, Javid et al. (2018) synthesised a number of hydrazone and indoline derivatives of 1,3,4-oxadiazole. They were examined for thymidine phosphorylase inhibition in comparison with 7-deazaxanthin. The most active from the hydrazone series was compound **28** (Figure 12) (approx. 30 times stronger inhibitor than the reference drug) and from the indolinone series compound **29** (Figure (12) (eight times stronger) [54,55].

**Figure 13:**
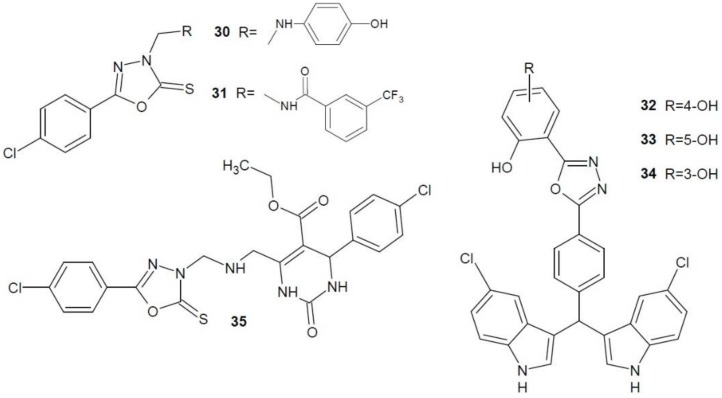
1,3,4-Oxadiazole derivatives with activity of thymidine phosphorylase inhibitors (part 2).

Bajaj et al. (2018) obtained a number of 5-(4-chlorophenyl)-1,3,4-oxadiazole-2-thione derivatives and tested them for anti-proliferative activity on the breast cancer cell line (MCF-7). The most active of them were **30** and **31** (Figure 13), whose action was closest to that of the reference adriamycin. Moreover, the structures were tested as thymidine phosphorylase inhibitors. Both proved to be much stronger enzyme inhibitors than 7-deazaxanthin [56].

A group of scientists under the direction of Taha (2018) obtained a number of 1,3,4-oxadiazoles derivatives containing (bis-5-chloro-indol-3-yl)methyl substituent. The most active were derivatives **32**, **33**, **34** (Figure 13) with different position of hydroxyl groups in the phenyl ring, which, in comparison with 7-deazaxanthin, showed 4–10 times stronger inhibitory effect on thymidine phosphorylase [57].

Iftikhar et al. (2018) developed a number of different substituted tetrahydropyrimidine-2-one derivatives, some of which contained 1,3,4-oxadiazole. The most active was compound **35** (Figure 13) which showed more than 30 times stronger inhibition of thymidine phosphorylase than the reference 7-deazaxanthin. Moreover, in vivo studies on antiangiogenic activity were carried out. This compound proved to be a strong angiogenesis inhibitor [58].

### 2.11. Thymidylate Synthase Inhibitors

Thymidylate synthase (TS) is a key enzyme in the processes of DNA replication, transcription and repair. It belongs to the group of methyltransferases and it catalyses the conversion of deoxyuridine monophosphate (dUMP) into deoxythymidine monophosphate (dTMP). This reaction produces thymidylic acid, which is a building block of DNA. Compounds which are structural analogues of thymidylate synthase substrates, such as 5-fluorouracil, combine with TS to form inactive complexes. This leads to the depletion of thymidylic acid in the cells, which results in the inhibition of their division and ultimately death [59,60]. DNA synthesis in cancer cells is higher than in normal cells; therefore, thymidylate synthase inhibitors constitute an important group of anti-cancer drugs.

**Figure 14:**
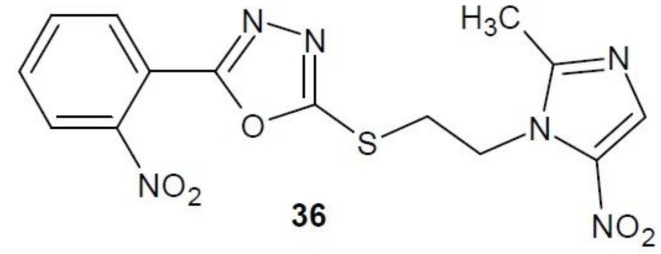
1,3,4-Oxadiazole derivative with activity of thymidylate synthase inhibitor.

In 2013 the results of research on 1,3,4-oxadiazole derivatives as inhibitors of thymidylate synthase were published. Anti-cancer activity was tested on three cell lines: liver cancer (HepG2), stomach cancer (SGC-7901) and breast cancer (MCF-7). The highest activity against liver cancer cells was observed in compound **36** (Figure 14). It turned out to be 30 times stronger than the reference 5-fluorouracil. In comparison to other cell lines, compound **36** and 5-fluorouracil showed comparable activity. The value of inhibitory concentration in the presence of human or bacterial thymidylate synthase (isolated from *Escherichia coli*) was also investigated. Compared to the reference raltitrexed, compound **36** had a similar effect on human TS and 10 times higher effect on bacterial TS [61].

### 2.12. 1,3,4-Oxadiazole Derivatives with Unknown Anti-Cancer Mechanism of Action

In many scientific studies, the anti-cancer activity of 1,3,4-oxadiazole derivatives against various in vitro cell lines has been described, without providing an exact mechanism of their action.

**Figure 15:**
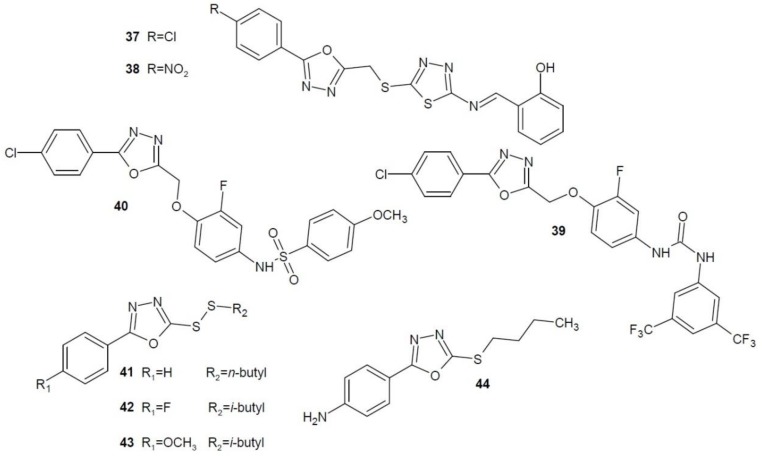
1,3,4-Oxadiazole derivatives with unknown anti-cancer mechanism of action.

Zhang et al. (2014) developed a number of derivatives of hybrid Schiff bases containing 1,3,4-oxadiazole and 1,3,4-thiadiazole rings. Their anti-proliferative activity was tested on several cancer cell lines: liver cancer (SMMC-7721), breast cancer (MCF-7), lung cancer (A549). The most active in liver cancer cells was derivative **37** (Figure 15), while compound **38** (Figure 15) had the strongest effect on breast and lung cancer cells. Both structures were two or three times stronger than the reference 5-fluorouracil [62].

Gamal El-Din et al. (2015) conducted research on diarylurea derivatives containing 1,3,4-oxadiazole ring in the structure. The obtained compounds were tested on many cancer cell lines. The most active compound **39** (Figure 15), compared to the reference sorafenib, showed stronger anti-proliferative effects against prostate (PC-3), colorectal (HCT-116), and kidney (ACHN) cancer cell lines [63].

In 2015, the same group of researchers also synthesised 1,3,4-oxadiazole derivatives containing the sulfonamide group. Of all the derivatives obtained, the highest activity was exhibited by compound **40** against breast cancer cell lines (T-47D and MDA-MB-468), SR leukaemia, melanoma (SK-MEL-5). The potency of derivate **40** was comparable to, or even higher than, the reference geftinib [64].

In 2016, Zhao et al. examined asymmetric disulphides connected to the 1,3,4-oxadiazole ring for anti-cancer activity. Studies were conducted on the cell lines of liver (SMMC-7721), cervix (HeLa) and lung cancer (A549). Compounds **41**, **42** and **43** (Figure 15) showed various degrees of anti-proliferative properties. All compounds strongly inhibited the growth of liver cancer cells (SMMC-7721) and had comparable activity against lung cancer (A549). Compound **41** showed the strongest inhibition of cervical cancer cells (HeLa). Derivatives **41**, **42**, **43** showed higher potency than the reference 5-fluorouracil [65].

A team of Indian scientists (2017) also synthesised new derivatives based on the structure of 1,3,4-oxadiazole. The most promising results of all the obtained compounds were found in derivative **44** (Figure 15). The study used the MTT ((3-(4,5-dimethylthiazol-2-yl)-2-5-diphenyltetrazolium bromide) cytotoxicity assay. This is a colorimetric method, which is the most commonly used method of measuring cytotoxicity and proliferation [66]. The test showed that compound **44** had a high inhibitory effect on breast cancer cells (MCF-7), stronger than the reference doxorubicin. At the same time, it was less toxic and safer with regard to normal cell lines (HEK-293). Flow cytometry analysis showed that cancer cells were arrested in the G0/G1 phase of the cell cycle. Western blotting studies (an electrophoresis-based method for the detection of specific proteins [67]) showed high activation of apoptotic protein caspase-3 and decreased expression of antiapoptotic protein Bcl-2 [68].

## 3. Summary

The conducted review of 1,3,4-oxadiazole derivatives shows that they have an anti-proliferative effect. These compounds have different mechanisms of action, which is very important in view of the observed resistance of tumours to standard drug treatment. They are inhibitors of growth factors, enzymes, kinases, and receptors such as the epidermal growth factor receptor (EGFR), vascular endothelial growth factor receptor (VEGFR), endothelin receptor (ET), histone deacetylase (HDAC), methionine aminopeptidase (MetAP), nuclear factor κB (NF-κB), poly(ADP-ribose) polymerase (PARP-1), telomerase enzyme, thymidine phosphorylase (TP), and thymidylate synthase (TS). The most active derivatives are more potent than the reference drugs already on the market, which proves the high potential of 1,3,4-oxadiazole derivatives as new medicinal substances. Further in vivo research is necessary to confirm efficiency of anti-proliferation activity and their safety.
